# Study on the influence of compaction energy and moisture content on the permeability and compression characteristics of granite-weathered soil

**DOI:** 10.1371/journal.pone.0352684

**Published:** 2026-07-02

**Authors:** Haibo Lyu, Dongcheng Wei, Jianxiao Gu, Liyun Fan

**Affiliations:** 1 College of Architecture and Electrical Engineering, Hezhou University, Hezhou, Guangxi, China; 2 College of Civil Engineering and Architecture, Guilin University of Technology, Guilin, Guangxi, China; 3 College of Architecture and Energy Engineering, Wenzhou University of Technology, Wenzhou, China; China University of Mining and Technology, CHINA

## Abstract

The feasibility of reusing granular weathered (GW) soil as foundation fill is closely related to the strength and stability. Initial moisture content and compaction energy were identified as the main factors affecting the strength and deformation behavior of GW soil. GW soil samples were prepared with different initial moisture contents ranging from 7 ~ 15% and compacted under varying compaction energies ranging from 50 ~ 125%, respectively. The results indicate that the permeability coefficient decreases sharply with increasing compaction energy and then gradually stabilizes at higher compaction energy levels, suggesting that compaction energy is the dominant factor governing soil permeability. Higher initial moisture content leads to larger strains due to the presence of thicker adsorbed water films, whereas higher compaction energy enhances interparticle bonding, reduces the void ratio, and increases soil stiffness. In particular, the void ratio is affected by both initial moisture content and compaction energy, highlighting the necessity of incorporating compaction energy into predictive permeability models. Furthermore, a compaction-energy-based permeability prediction model was developed, with the predicted results showing strong agreement with the experimental data. These findings provide practical guidance for optimizing the compaction of GW soil to enhance foundation stability and reduce the risks associated with rainfall infiltration, and seepage failure.

## 1. Introduction

The rapid expansion of infrastructure and urbanization has substantially increased the demand for fill materials, leading to the widespread use of locally sourced and reprocessed soils as engineering fill in construction practice [1–5]. Consequently, the engineering performance of fill soils is critical to the serviceability, durability, and long-term safety of geotechnical structures. Inadequate control of compaction degree and moisture content may result in excessive deformation, differential settlement, and seepage, which can ultimately compromise structural stability under environmental actions such as rainfall infiltration and groundwater-level fluctuations. Numerous studies have shown that both moisture content and dry density have pronounced effects on key soil properties, including shear strength, stiffness, and permeability. Existing research has primarily focused on the effects of molding moisture content, chemical additives, dry density, and compaction conditions on soil mechanical behavior, including shear strength, cyclic resistance, deformation response, and volumetric change behavior [6–12]. However, most existing studies primarily focus on mechanical responses, whereas permeability behavior and its coupling with compaction energy remain inadequately investigated. While permeability is typically derived indirectly from void ratio or dry density, compaction energy—despite being directly controllable during field construction—remains largely absent from existing permeability prediction models. As a result, the applicability of existing empirical relationships in engineering practice is limited. Therefore, this study investigates the behavior of GW soil under varying compaction energies and initial moisture contents, with particular emphasis on permeability evolution, compressibility, and deformation behavior.

In recent decades, various algorithms have been employed to investigate the complex relationships between soil properties and water content [13–17]. For instance, Chen et al. [18] investigated the effects of moisture content on the shear strength and microstructure of remolded soil samples. Sun et al. [19] demonstrated that the shear strength of compacted bentonite is influenced by moisture content during aging, reaching a minimum at a moisture content of 24%. Moreover, Bakhshi et al. [16] showed that soil pore geometry significantly affects both water retention and soil-water characteristic curve (SWCC) patterns. These studies did not explicitly consider the effects of compaction energy and initial moisture content on soil permeability and compressibility. Moreover, microstructural characterization techniques—including mercury intrusion porosimetry (MIP), scanning electron microscopy (SEM), nuclear magnetic resonance (NMR), and X-ray imaging—have been widely employed to elucidate the relationship between soil moisture content and mechanical properties [18–22]. Although the aforementioned studies have provided valuable insights, the effects of compaction energy and moisture content on the mechanical behavior of GW soil remain insufficiently investigated and require further experimental study.

Both laboratory permeability tests and field observations indicate that compaction energy and initial moisture content significantly influence soil mechanical behavior. Nevertheless, studies that explicitly examine their effects on soil permeability remain limited [23–25]. For instance, Zhang et al. [26] reported that, in consolidation tests on compacted loess, initial moisture content and porosity were positively correlated with compressibility and negatively correlated with compressive yield stress. Uyeturk and Huvaj [27] demonstrated through direct shear tests on compacted residual soils that soil shear strength increases with decreasing degree of saturation. Similarly, Yang et al. [28] revealed through consolidation tests that moisture content significantly influences the void ratio and secant modulus of loess. With increasing moisture content, the relationship between void ratio and moisture content transitions from linear to nonlinear. Although previous studies have investigated the effects of compaction energy and moisture content [10–11], the underlying mechanisms governing the behavior of GW soil remain inadequately characterized. This limitation primarily arises from difficulties in comprehensively elucidating the coupled relationships among compaction energy, initial moisture content, and mechanical strength, thereby constraining the application of GW soil as a subgrade fill material.

This study investigates the permeability and compressibility characteristics of GW soil under varying compaction energies and initial moisture contents. A series of laboratory permeability and consolidation tests were conducted on samples prepared at initial moisture contents of 7%, 9%, 11%, 13%, and 15%, and compacted at energy levels corresponding to 50%, 75%, 100%, and 125% of the standard heavy compaction energy. The test results were used to analyze the relationships between compaction energy and permeability coefficient, void ratio and consolidation pressure, consolidation pressure and compressive strain, consolidation pressure and compression coefficient, and consolidation pressure and compression modulus. Furthermore, a compaction-energy-based permeability model was developed to evaluate the combined effects of compaction energy and initial moisture content on GW soil permeability. This study provides valuable insights for infrastructure design, particularly for the application of GW soil as a subgrade fill material.

## 2. Materials and sample preparation

### 2.1 Soil properties

**Fig 1:**
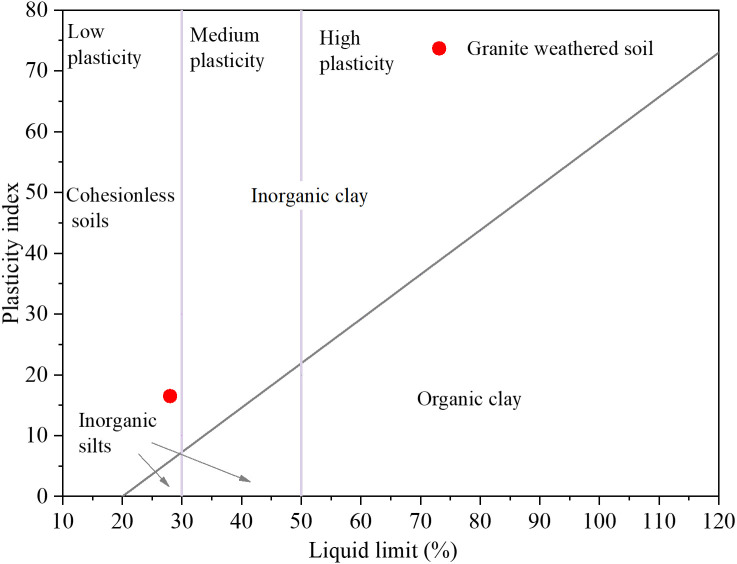
Soil plasticity chart from Unified Soil Classiﬁcation System. Granite weathered soil: low plasticity cohesionless soils.

The soil samples used in the experiments was granite weathered soil (GW soil) collected from a mountainous construction site located at the boundary between Guiling Town and Daning Town, Hezhou, Guangxi, China. The soil exhibited grayish-yellow to brown coloration and showed a tendency to soften and disintegrate upon immersion in water. To fully utilize these GW soils, their potential application as subgrade fill materials is being explored. Prior to implementation, it is essential to characterize the compaction behavior and water retention properties of the soil samples. After natural air-drying, the GW soil samples were gently crushed with a rubber mallet and sieved through a 2 mm (No. 10) mesh to remove stones and impurities. The prepared samples were then sealed in plastic bags for later use. The basic physical properties of the GW soil used in the experiments are listed in Table 1. The specific gravity was determined in accordance with ASTM (2023) [29], the liquid and plastic limits were determined following ASTM (2017) [30], and the plasticity index was calculated as the difference between the liquid limit and the plastic limit. The soil has a specific gravity of 2.69, a liquid limit of 28%, a plastic limit of 11.5%, and a plasticity index of 16.5%. Based on the Unified Soil Classification System (USCS), the GW soil tested in this study is classified as low plasticity cohesionless soils, as shown in Fig 1.

**Table 1:** Physical properties of granite weathered soil from Hezhou.

Index	value	
Speciﬁc gravity *G*_s_	2.69	
Liquid limit *W*_L_（%）	28.0	
Plastic limit *W*_p_（%）	11.5	
Plasticity index *I*_p_	16.5	
Compaction Energy	Optimum moisture content	Maximum dry density
50%	11.53%	1.85 g/cm^3^
75%	11.24%	1.89 g/cm^3^
100%	10.82%	1.91 g/cm^3^
125%	10.56%	1.96 g/cm^3^

**Fig 2:**
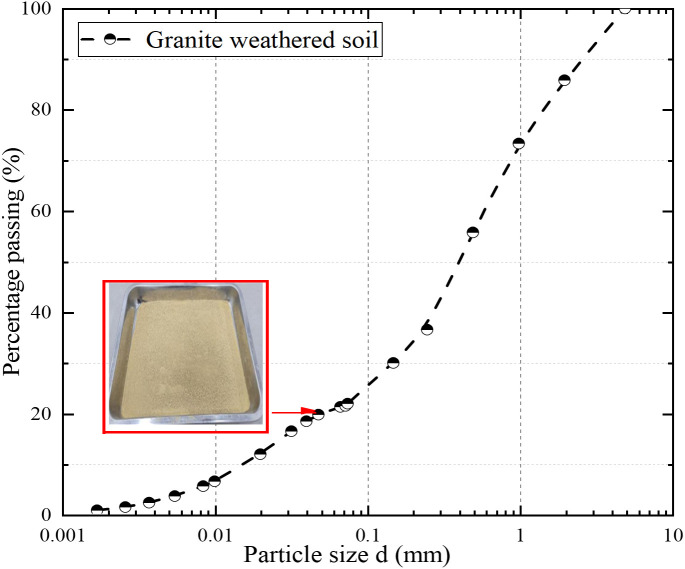
Grain size distribution curves for granite weathered soil.

Moreover, Fig 2 presents the particle size distribution of the GW soil, which exhibits a broad gradation, with particle sizes ranging from clay-sized particles to gravel-sized fractions and a relatively smooth gradation curve. These characteristics contribute to the favorable engineering performance of GW soil, particularly its low liquefaction potential and excellent compaction behavior. The soil samples used in this study were collected from publicly accessible construction materials and no specific permits were required for sample collection. The study did not involve protected areas, endangered species, or privately restricted land.

### 2.2 Sample preparation

**Fig 3:**
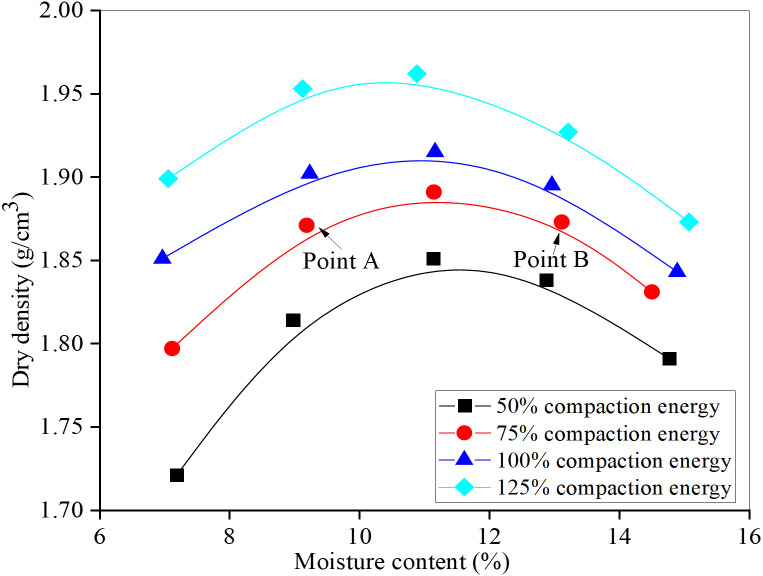
Dry density distribution under different sample preparation conditions.

Compaction degree is one of the key indicators for evaluating the quality of soils used as backfill materials, and it is primarily influenced by dry density and moisture content. Therefore, to investigate the permeability and compressibility characteristics of GW soil used as fill material, samples with different moisture contents (7%, 9%, 11%, 13%, and 15%) were prepared and subjected to heavy compaction tests. Compaction tests were conducted on four groups of samples using a JDS-1 electric digital compaction instrument (produced by Nanjing Soil Instrument Factory). The applied compaction energies were set at 50%, 75%, 100%, and 125% of the standard heavy compaction energy, equating to1341 kJ/m³ (E1), 2011.5 kJ/m³ (E2), 2682 kJ/m³ (E3), and 3352.5 kJ/m³ (E4), respectively. Fig 3 shows the variation in dry density of GW soil with initial moisture content under different compaction energies. It is observed that, at a given compaction energy, the dry density first increases and then decreases with increasing moisture content. The moisture content and dry density corresponding to the peak of each compaction curve are defined as the optimum moisture content and maximum dry density, respectively, for that compaction energy. Moreover, with increasing compaction energy, the compaction curves shift toward the upper-left direction. At low moisture contents (<10%), an increase in compaction energy results in a pronounced increase in dry density. As summarized in Table 1, the optimum moisture content decreases, whereas the maximum dry density increases with increasing compaction energy. This behavior can be attributed to the higher compaction energy, which overcomes frictional and cohesive forces between soil particles and allows them to be rearranged into a denser configuration even at relatively low water contents.

**Fig 4:**
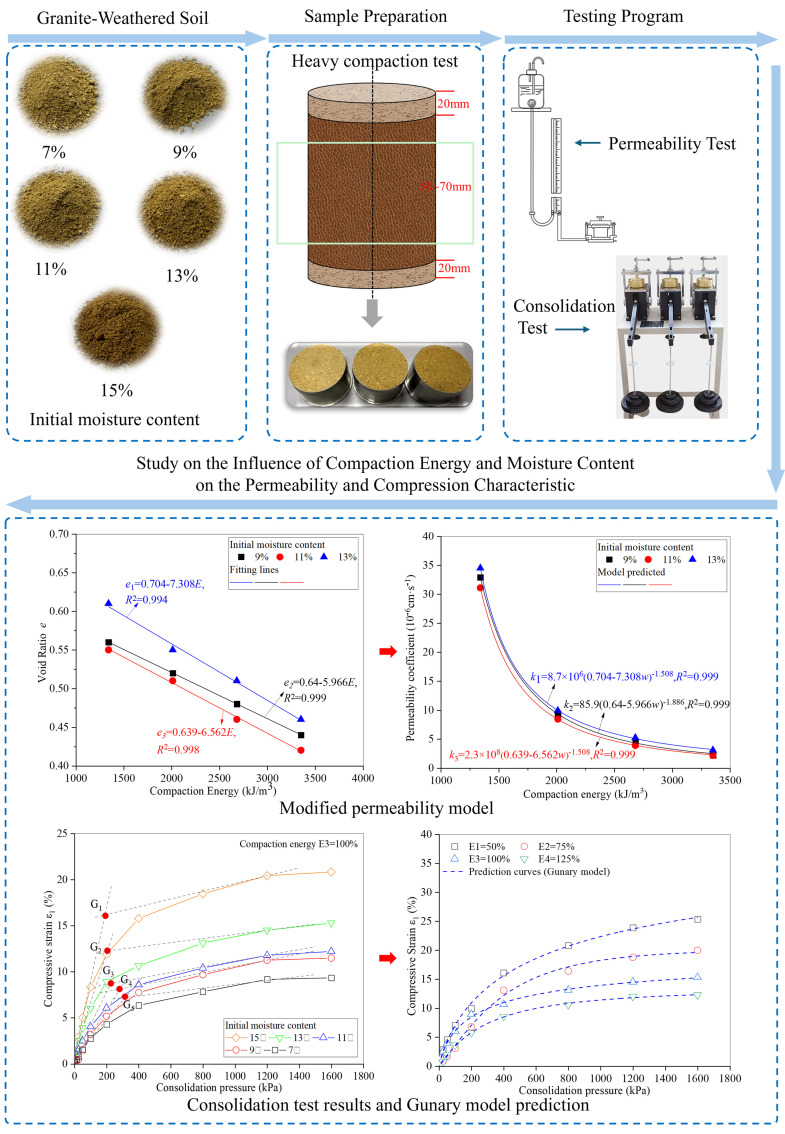
Flow diagram of preparation and testing process of samples in this study.

Moreover, Fig 3 shows that the moisture content versus dry density curve has a unimodal form, allowing samples with identical dry densities to be achieved at different moisture contents. Dry density partially reflects the samples’s pore structure, which significantly influences its mechanical properties. For each test condition, three parallel samples were prepared, and the mean value was reported [31]. Data points with deviations exceeding 15% from the mean were excluded, and the tests were repeated to ensure data reliability [32]. The ring-cutter samples used in the tests were prepared from samples after compaction testing, following the procedure described below. Each compact sample was cut along its vertical central axis to minimize disturbance. Approximately 20 mm of material was removed from the top (affected by hammer impact) and the bottom (abnormally densified due to contact with the base plate) in accordance with standard procedures. A representative region was then selected from the remaining middle section (approximately 50–70 mm in height) for sampling, as illustrated in Fig 4. All GW soil samples used for the one-dimensional consolidation and permeability tests were sampled from the middle section of the compacted samples (approximately 50–70 mm in height) to ensure specimen uniformity. Moreover, the prepared samples were immediately sealed with plastic wrap and cured in a humidity-controlled chamber for 72 h. Subsequently, the samples were vacuum-saturated for 2 h and then immersed in distilled water prior to testing.

## 3. Testing program

### 3.1 One-dimensional consolidation test

The samples for one-dimensional consolidation test were 61.8 mm in diameter and 20 mm in high and shaped into a cylindrical ring. The vertical consolidation stresses were designed as follows: 12.5, 25, 50, 100, 200, 400, 800, 1000, and 1600kPa. Generally, each loading was imposed for 24 hours until the settlement due to the previous load has ceased, or the displacement rate was lower than 0.01 mm/h [32–33]. The experimental program of one-dimensional consolidation test shows in Table 2, and the recorded value of initial moisture contents, and void ratio is a mean value of all samples in the same condition.

**Table 2:** Test scheme design for granite weathered soil considering water content and compaction energy.

Type	Initial Moisture Contents (%)	Compaction Energy (kJ/m^3^)	Number of Compaction Layers	Blows Per Layer
Compaction Test	7, 9, 11, 13, 15	1341	5	14
		2011.5		20
		2682		27
		3352.5		34
Permeability Test	9, 11, 13	1341	_	_
		2011.5		
		2682		
		3352.5		
One-Dimensional Consolidation Test	11	1342, 2011.5, 2682, 3352.5	_	_
	7, 9, 11, 13, 15	2682		

### 3.2 Permeability test

The permeability test was conducted in accordance with the Standard for Soil Test Methods. The samples for permeability test were 61.8 mm in diameter and 40 mm in high and shaped into a cylindrical ring. Then, after sample preparation, the sample was placed in the permeameter, and both the top and bottom surfaces were saturated using de-aired water under a low hydraulic gradient. Air was carefully expelled from the system to avoid trapped bubbles. When stable saturation was achieved, the permeability test was performed under a constant (or falling) hydraulic head condition. The inflow and outflow volumes were recorded at predetermined time intervals until a steady-state flow was established. The coefficient of permeability was then calculated based on Darcy’s law. The initial moisture contents for the permeability test were set to 9%, 11%, and 13%. For each moisture content, samples compacted under four compaction energy levels (50%, 75%, 100%, and 125%) were prepared. The detailed testing program is summarized in Table 2.

## 4. Results and discussion

### 4.1 The effect of compaction energy on permeability

**Fig 5:**
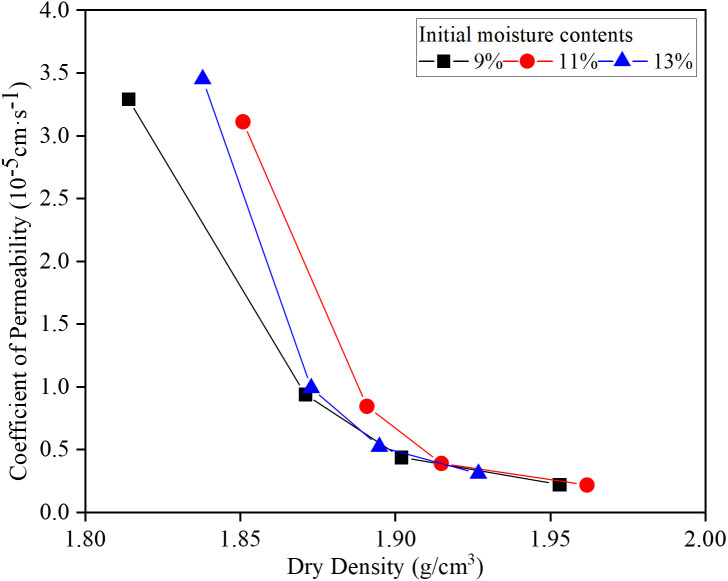
Relationship between permeability coefficient and dry density at different initial moisture contents.

As shown in Table 1 and Fig 3, the effect of different compaction energies on GW soil can be represented by dry density; that is, higher compaction energy leads to greater dry density. Under compaction energies of 50%, 75%, 100%, and 125%, samples with dry densities of 1.85, 1.89, 1.91, and 1.96 g/cm³, respectively, are obtained. Fig 5 illustrates the variation in permeability coefficient with dry density for samples prepared at different initial moisture contents. The results indicate that both dry density and initial moisture content influence the permeability coefficient of the GW soil samples. As dry density (compaction energy) increases, the permeability coefficient initially decreases rapidly and then more gradually, eventually approaching a stable value. At low dry densities (compaction energies), the permeability coefficient remains relatively high and is essentially independent of initial moisture content. The permeability coefficients of GW soil samples with initial moisture contents of 9%, 11%, and 13% are 3.29 × 10 ⁻ ⁵, 3.11 × 10 ⁻ ⁵, and 3.45 × 10 ⁻ ⁵ cm·s ⁻ ¹, respectively. With further increases in dry density (compaction energy), the permeability coefficient decreases sharply. When the dry density becomes sufficiently high, that is, when adequate compaction energy (compaction energy 125%) is applied during sample preparation, the permeability coefficient gradually approaches an approximately constant value of about 2 ~ 3 × 10 ⁻ ^6^ cm·s ⁻ ¹. At the same initial moisture content, the permeability coefficient of the GW soil sample compacted at 50% compaction energy is 11–14 times greater than that of the sample compacted at 125% compaction energy. These results indicate that dry density (compaction energy) is the dominant factor controlling sample permeability.

At low dry densities (compaction energies), the samples contain relatively large pores with good connectivity. As dry density (compaction energy) increases, the void ratio decreases and the number of effective flow channels is reduced, thereby hindering water migration and ultimately leading to a lower permeability coefficient. At high dry densities (compaction energies), corresponding to higher compaction energies, the permeability coefficient exhibits pronounced sensitivity to dry density under different initial moisture contents. This observation indicates that the permeability of densely compacted GW soil is highly sensitive to changes in dry density. Moreover, previous studies have also demonstrated that the permeability coefficient decreases markedly with increasing dry density and degree of compaction, particularly under highly compacted conditions [34]. Therefore, when GW soil is used as a foundation fill material, its dry density should be increased to values greater than 1.9 g/cm³. At this density level, soil particles are more densely packed, and the permeability–dry density curve becomes nearly flat, effectively mitigating the adverse effects of rainfall infiltration and groundwater erosion, reducing the risks of differential settlement and seepage failure, and enhancing the long-term stability and safety of engineering structures.

**Fig 6:**
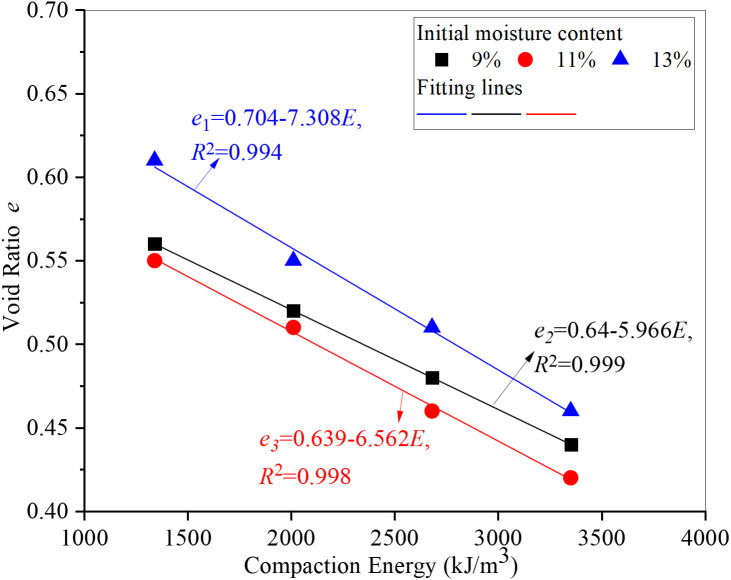
Relationship between compaction energy and void ratio.

The void ratio is one of the primary factors governing the permeability coefficient. The void ratio is governed by multiple factors, including depositional conditions, particle characteristics, stress state, and environmental effects, which collectively determine the proportion of pore volume relative to solid particle volume in soils or rocks. In compacted geomaterials, the void ratio is closely linked to dry density and compaction energy, such that increasing compaction energy generally leads to a reduction in void ratio and a more compact particle arrangement. At low dry densities (low compaction energies), soils typically exhibit relatively large and well-connected pores, providing abundant flow channels and thus higher permeability. Previous studies have demonstrated that this permeability reduction is primarily driven by pore structure refinement induced by compaction [6]. For compacted fill soils, the stress state (i.e., compaction energy) is the dominant factor controlling the post-compaction properties. Therefore, it is necessary to establish explicit relationships among compaction energy, moisture content, and permeability coefficient to directly characterize the permeability behavior of granular weathered (GW) soil. Mesri et al. [35] experimentally proposed an empirical relationship between the permeability coefficient and the void ratio for cohesive soils, as expressed in Equation (1). This relationship is widely interpreted as evidence that permeability is fundamentally governed by pore space geometry rather than compaction variables themselves [6]. However, neither study directly established the relationship between compaction energy and moisture content and the permeability coefficient. Compaction conditions are considered to influence permeability indirectly by modifying the void ratio and pore structure, which serve as the key intermediate variables linking compaction state and hydraulic conductivity.


$$\begin{array}{c} k=Be^{A} \end{array}$$ (1)

where A and B denote permeability-related parameters, which are obtained by fitting the data from consolidation–permeability tests. To investigate the relationship between compaction energy and permeability, the test results are presented in Fig 6 and Table 3. The results indicate that the void ratio of GW samples decreases linearly with increasing compaction energy.

**Table 3:** Permeability test results of GW soil.

Moisture content (%)	Compaction Energy (kJ/m^3^)	Void Ratio	Permeability Coefficient (10^−6 cm^·s^-1^)
9	1341	0.56	32.9
	2011.5	0.52	9.39
	2682	0.48	4.37
	3352.5	0.44	2.21
11	1341	0.55	31.1
	2011.5	0.51	8.43
	2682	0.46	3.88
	3352.5	0.42	2.16
13	1341	0.61	34.5
	2011.5	0.55	9.89
	2682	0.51	5.21
	3352.5	0.46	3.07

In addition, when the initial moisture content is close to the optimum value, the samples exhibit the minimum void ratio, regardless of compaction energy. Notably, samples with the same dry density can still exhibit different void ratios (points A and B in Fig 3), indicating that void ratio alone is insufficient to characterize permeability. Therefore, the effect of compaction energy should be explicitly incorporated into the permeability prediction model. The relationship between compaction energy and void ratio is described by Equation (2):


$$\begin{array}{c} e=a+bE \end{array}$$ (2)

Here, E represents the compaction energy, and a and b are empirical fitting parameters that depend on the soil’s deposition conditions, particle characteristics, stress state, and environmental factors.

By substituting Equation (2) into Equation (1), a compaction-energy-based model for predicting permeability can be obtained, as presented in Equation (3).


$$\begin{array}{c} k=B(a+bE{)}^{A} \end{array}$$ (3)

**Fig 7:**
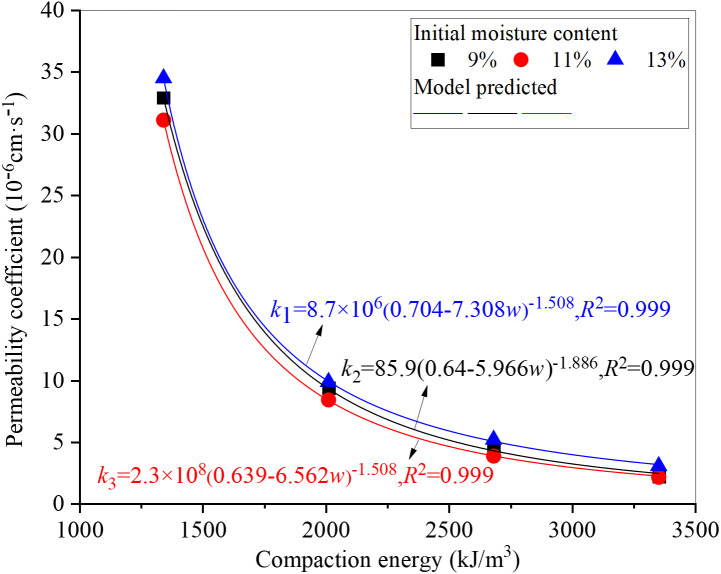
Comparison between the model-predicted values and the experimental results.

To validate the reliability of the modified model, Fig 7 compares the predicted values with the experimental data. The results show that the model predictions closely follow the overall trend of the measured data, demonstrating a high goodness of fit. These results indicate that the modified model enables direct estimation of soil permeability based on compaction energy, providing a more practical and application-oriented alternative to traditional empirical relationships that rely primarily on void ratio or indirect state variables.

### 4.2 The effect of compaction energy on mechanical behavior

**Fig 8:**
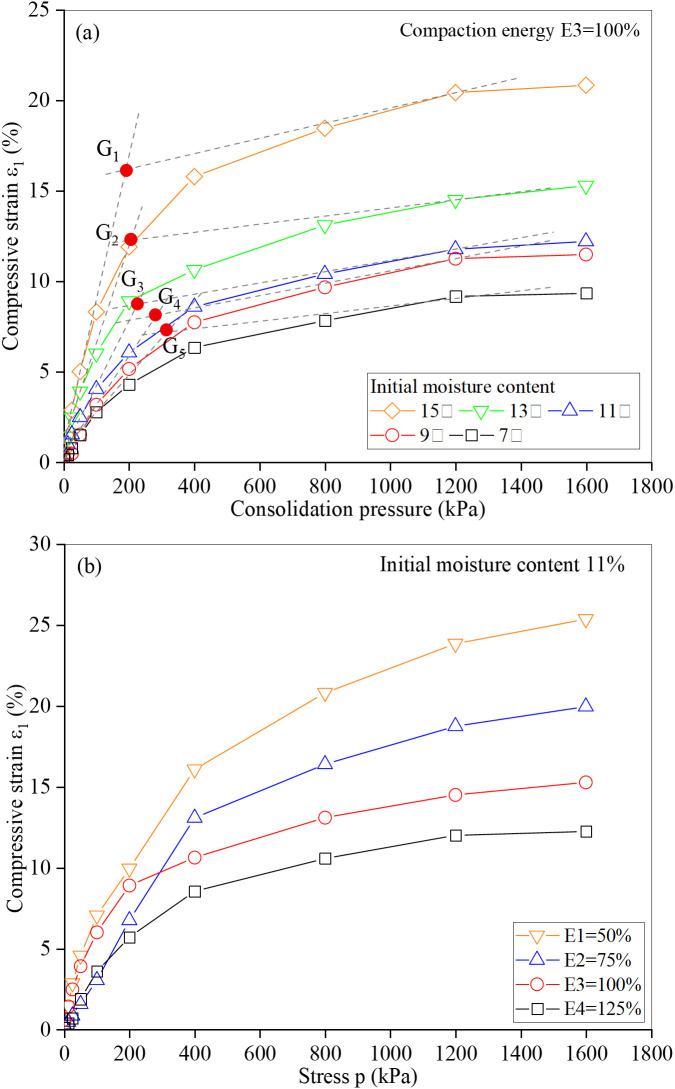
Relationship between compressive strain and vertical stress of GW soil under different conditions. **(a)** Compaction energy 2682 kJ/m^3^, and **(b)** Initial moisture content 11%.

Fig 8(a) shows the stress–strain curves of samples with varying initial moisture contents under 100% compaction energy. As stress increases, strain also increases, while the strain rate gradually decreases. A distinct inflection point is observed in the strain rate, beyond which it tends to stabilize. For the sample with an initial moisture content of 15%, the inflection point on its stress–strain curve (corresponding to point G₁ in Fig 8(a)) occurs at a stress of approximately 200 kPa and a strain of about 16%. At low stress levels, all samples exhibited significant deformation. Moreover, at the same stress level, samples with higher initial moisture content exhibited greater strain than those with lower moisture content. For instance, at a stress of 200 kPa, the strain of the sample with an initial moisture content of 15% was approximately 16%, whereas that of the sample with an initial moisture content of 7% was only about 5%, indicating that moisture content has a significant effect on soil structure. This behavior is mainly attributed to the development of thicker adsorbed water films surrounding soil particles at higher moisture contents, which reduce matric suction and weaken interparticle cohesion. Moreover, the increase in water film thickness not only attenuates interparticle bonding and reduces the stiffness of the soil skeleton, but also enhances lubrication at particle contacts, thereby facilitating relative particle sliding and particle rearrangement. Consequently, more compressible pore space is retained within the soil, ultimately resulting in larger deformation.

For samples with a moisture content of 11%, the stress–strain relationships under different compaction energies are shown in Fig 8(b). The compressive strain increased monotonically with increasing vertical stress, while the strain increment rate gradually declined. At relatively low stress levels, the samples exhibited significant deformation. Under the same stress, samples compacted with lower initial energy experienced larger strains than those compacted with higher energy. For instance, at a vertical stress of 200 kPa, the strain of the sample with an initial compaction energy of 50% was approximately 10%, whereas that of the sample with 125% was only about 5%. These results indicate that initial compaction energy significantly influences soil structure. This behavior can be attributed to the denser particle arrangement under higher initial compaction energy, resulting in higher interparticle effective stress and a stiffer soil skeleton. Moreover, higher initial compaction energy enhances interparticle bonding and interlocking, thereby increasing the deformation resistance of the soil skeleton. It also pre-compresses the internal pore space, significantly reducing the remaining compressible volume and ultimately leading to smaller deformations. Based on previous studies, various mathematical functions can be used to fit the stress–strain relationship of remolded soils under unconfined compression. In this study, the Gunary model was selected to analyze the unconfined compressive stress–strain behavior of decomposed granite soil under varying initial compaction energies. The mathematical expression is given as follows [36]:


$$\begin{array}{c} \varepsilon_{si}=P_{i}/\left(a_{\text{1}}+b_{\text{1}}P_{i}+c_{\text{1}}\sqrt{P_{i}}\right) \end{array}$$ (4)

Here, $P_{i}$ denotes the applied vertical stress at each loading stage in the compression test, $\varepsilon_{si}$ represents the total axial strain of the sample under unconfined compression at each loading level, and $a_{\text{1}}$, $b_{\text{1}}$, and $c_{\text{1}}$ are fitting parameters obtained from experimental data under a given compaction degree and initial water content.

**Fig 9:**
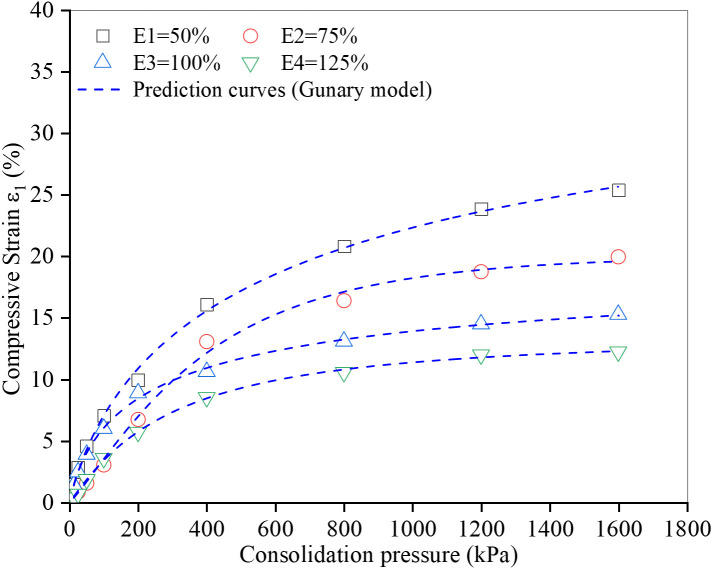
Comparison between Gunary model predictions and experimental results under different compaction energies.

Equation (4) was used to fit the consolidation compression results of GW soil under varying initial compaction conditions, with the fitting results summarized in Table 4. As shown in Table 4 and Fig 9, the stress–strain behavior of compacted GW soil under unconfined conditions is well described by the Gunary model. The coefficients of determination (R^2^) all exceed 0.996, indicating excellent agreement between the fitted curves and experimental results. The fitted curves closely reproduce the evolution trends of the measured stress–strain responses, demonstrating the model’s capability to accurately capture the experimental behavior. At the same consolidation pressure, samples with lower initial moisture content exhibit a larger void ratio than those with higher moisture content. For instance, at a consolidation pressure of 100 kPa, the void ratio of the sample with an initial moisture content of 7% is approximately 0.46, compared with approximately 0.45 for the sample with 15% moisture content. At the same consolidation pressure, samples with lower initial moisture content exhibit higher void ratios than those with higher moisture content. For instance, at a consolidation pressure of 100 kPa, the void ratio of the sample with an initial moisture content of 7% is approximately 0.46, compared with approximately 0.45 for the sample with 15% moisture content.

**Table 4:** Fitting results of the stress–strain relationship for GW soil.

Type	Compaction Energy (kJ/m^3^)	Dry density (g/cm^3^)				R^2^
			$a_{1}$	$b_{1}$	$c_{1}$	
GW soil	1341	1. 85	7.977	0.023	0.476	0.998
	2011.5	1. 89	36.678	0.066	−1.504	0.996
	2682	1. 91	5.87	0.047	0.587	0.997
	3352.5	1. 96	26.532	0.078	−0.521	0.999

When the consolidation pressure increases to 1000 kPa, the corresponding void ratios decrease to approximately 0.38 and 0.37, respectively. These results indicate that moisture content plays a significant role in controlling the pore structure and compressibility of the soil. This behavior can be attributed to differences in the role of water at different moisture contents. At lower moisture contents, thinner water films form between soil particles, resulting in reduced lubrication and diminished pore-filling effects. Consequently, the particles remain more loosely arranged, leading to a lower overall density and stiffness of the soil skeleton. In contrast, at excessively high moisture contents, the structural strength of the soil is significantly weakened by water, and interparticle bonding is markedly reduced. Meanwhile, abundant water occupies part of the micropores, causing partial pre-compression of the internal pore space and limiting the soil’s subsequent compressibility, which ultimately results in a smaller void ratio.

**Fig 10:**
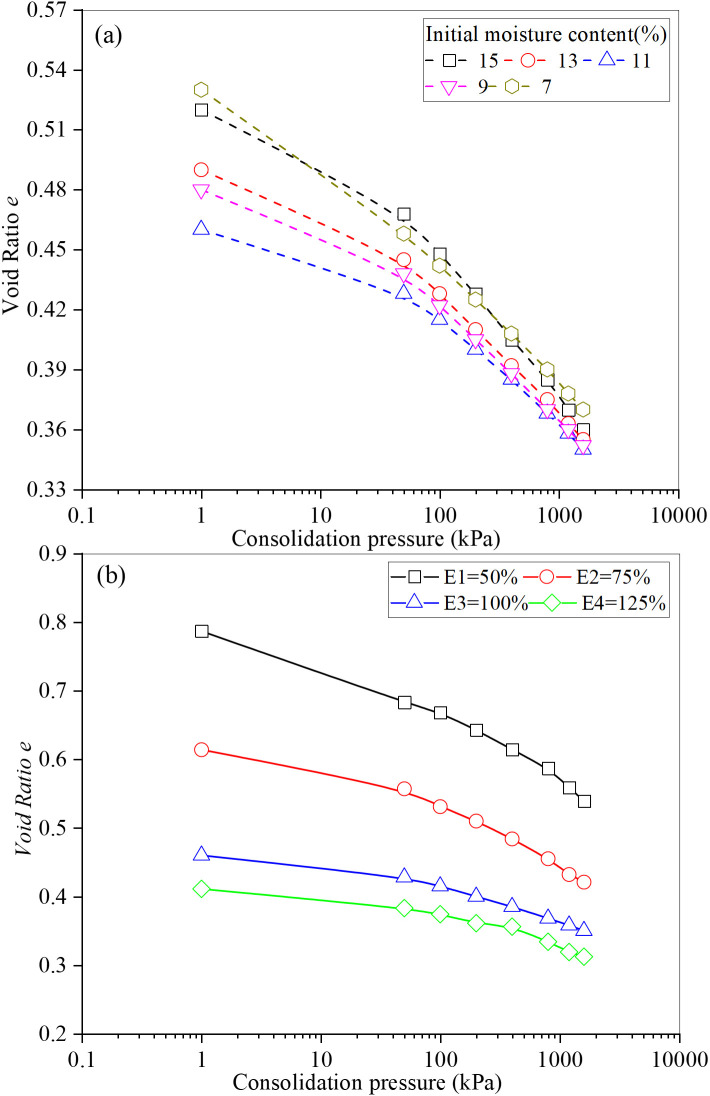
Void ratio–consolidation pressure relationships of GW soil under different initial conditions.

Fig 10(b) shows the void ratio–stress relationships of samples with 11% moisture content prepared under varying compaction energies. The void ratio decreases monotonically with increasing applied stress. Under the same applied stress, samples prepared with lower initial compaction energy exhibited higher void ratios than those prepared with higher compaction energy. For instance, at a consolidation pressure of 100 kPa, the void ratio of the sample compacted at 50% compaction energy was approximately 0.70, whereas that of the sample compacted at 125% compaction energy was only about 0.38. These results indicate that initial compaction energy plays a crucial role in regulating the pore structure of the soil. This behavior can be attributed to the denser arrangement of soil particles induced by higher initial compaction energy, which pre-compresses the internal pore space and results in a smaller initial void ratio. Moreover, higher compaction energy enhances particle interlocking and cementation, thereby increasing the stiffness of the soil skeleton. Consequently, the available space for further pore compression under subsequent loading is greatly restricted, ultimately resulting in a lower void ratio.

### 4.3 The effect of compaction energy on compression characteristics

**Fig 11:**
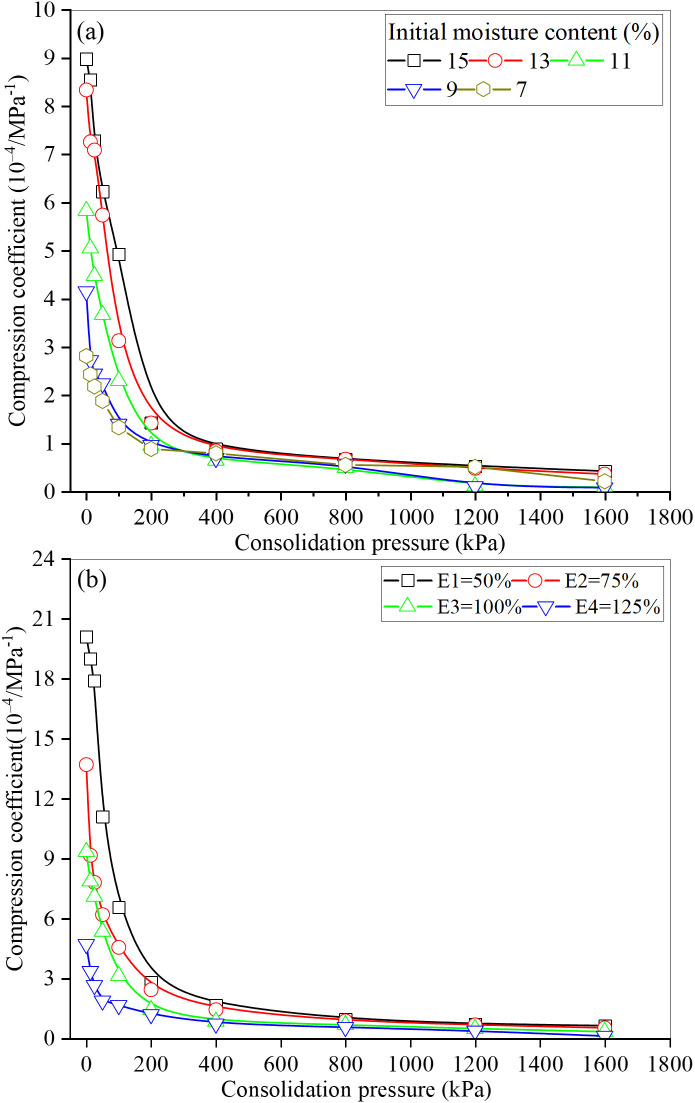
Relationship between compression coefficient and consolidation pressure of GW soil.

Under a compaction energy of 100%, the relationship between the compression coefficient and consolidation pressure is shown in Fig 11(a). For all initial moisture contents, the compression coefficient decreased sharply with increasing consolidation pressure and then gradually approached a nearly constant value. As shown in Fig 11(a), when the consolidation pressure was below 200 kPa, the compression coefficient of the soil decreased sharply. Once the pressure exceeded 400 kPa, the decrease rate slowed markedly, and the compression coefficients corresponding to different moisture contents gradually converged. Under the same consolidation pressure, soils with lower moisture contents exhibited smaller initial compression coefficients, indicating a significant influence of moisture content. Specifically, the initial compression coefficient of the sample with a moisture content of 15% was approximately 9 × 10 ⁻ ⁴, whereas that of the sample with a moisture content of 7% was only about 2.8 × 10 ⁻ ⁴. This behavior can be attributed to variations in pore water conditions. At low moisture contents, strongly bound water dominates, forming thin water films with high viscous resistance. As a result, significant compression occurs only under relatively high loads, leading to a smaller initial compression coefficient. In contrast, at higher moisture contents, abundant free water reduces interparticle friction and facilitates particle sliding and rearrangement, leading to rapid compression under loading and a larger initial compression coefficient.

For samples with 11% moisture content, the relationship between the compression coefficient and consolidation pressure is shown in Fig 11(b). For all compaction energies, the compression coefficient decreases sharply at first with increasing consolidation pressure and then gradually stabilizes. Specifically, the curves are steep at consolidation pressures below 200 kPa and gradually flatten as the pressure increases further. Additionally, at the same consolidation pressure, the initial compression coefficient is negatively correlated with compaction energy. For instance, the initial compression coefficient is approximately 2.0 × 10 ⁻ ³ for samples compacted at 50% and about 5.0 × 10 ⁻ ⁴ for those compacted at 125%. This is because low compaction energy results in loosely arranged soil particles with unstable arch structures and large voids, which are rapidly compressed under loading, leading to a higher compression coefficient. Conversely, at high compaction energy, soil particles are densely packed, forming a high dry-density, low-void framework. Particle mobility is limited, and only large loads can induce minor deformation, resulting in a lower compression coefficient.

**Fig 12:**
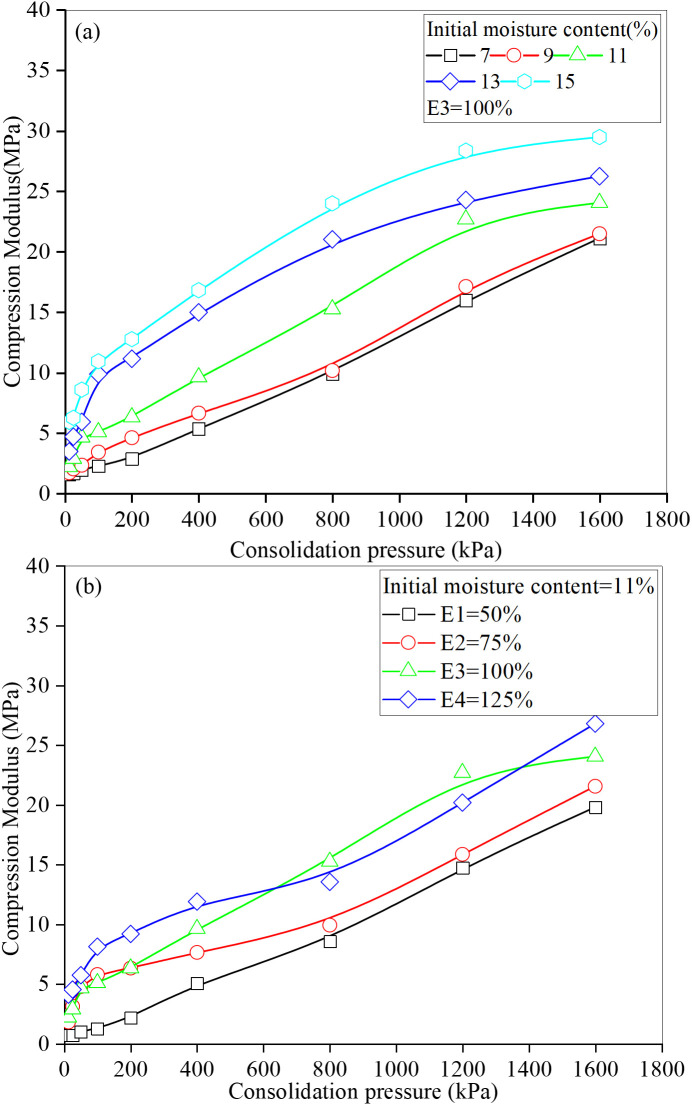
Test results under different water contents and varying compaction efforts.

Fig 12(a) presents the variation of the compression modulus with consolidation pressure for samples compacted at 100% compaction energy. For all moisture contents, the compression modulus increases monotonically with increasing consolidation pressure, while the rate of increase gradually decreases at higher pressures. At the same pressure, samples with lower moisture content exhibit higher compression modulus. For instance, at 1800 kPa, the modulus is approximately 30 MPa for the 7% moisture content sample, compared to about 20 MPa for the 15% moisture content sample. This behavior is attributed to the effect of moisture content. At low moisture content, thin water films separate soil particles, resulting in higher viscous resistance, denser particle packing, and increased stiffness of the soil skeleton. Consequently, the soil undergoes smaller deformations under load, leading to a higher compression modulus. Conversely, at high moisture content, abundant free water facilitates particle sliding, reduces the skeleton’s resistance to deformation, and lowers the compression modulus, highlighting the strong influence of moisture content on soil stiffness.

Fig 12(b) shows the variation of the compression modulus with pressure for samples with a moisture content of 11%. For all compaction energies, the compression modulus increases monotonically with increasing pressure, with the rate of increase gradually slowing at higher pressures. At the same pressure, higher compaction energy results in a higher compression modulus. For instance, at 1800 kPa, samples compacted at 3352.5 kJ/m³ reach approximately 28 MPa, while those compacted at 1341 kJ/m³ only reach about 20 MPa. This effect is due to the influence of compaction energy: at high compaction, soil particles are densely packed and voids are effectively compressed, resulting in a stiffer skeleton and reduced deformation under load, thereby increasing the compression modulus. In contrast, at low compaction, particles are loosely arranged with larger voids, the skeleton exhibits weaker resistance to deformation, and the compression modulus is consequently lower. This demonstrates the reinforcing effect of compaction energy on soil stiffness.

## 5. Conclusion

This study systematically investigated the effects of initial moisture content and compaction energy on the permeability and compressive behavior of GW soil. Laboratory permeability and consolidation tests were conducted for initial water contents of 7 ~ 15%, and compaction energies corresponding to 50 ~ 125% of the standard heavy compaction energy. The relationships among compaction energy and permeability coefficient, void ratio and consolidation pressure, consolidation pressure and compression strain, consolidation pressure and compression coefficient, and consolidation pressure and compression modulus were analyzed. A permeability model was developed to describe the relationship between compaction energy and the soil’s permeability. The results indicate that the modified model can directly predict soil permeability based on compaction energy, making it more practical and convenient than traditional empirical formulas. Based on the findings of this study, the following detailed points are concluded:

(1) The permeability of GW soil is governed primarily by compaction energy rather than initial moisture content. Once dry density exceeds approximately 1.9 g/cm³ (compaction energy 100%), the permeability coefficient stabilizes at 2 ~ 3 × 10 ⁻ ⁶ cm·s ⁻ ¹, effectively suppressing rainfall infiltration and groundwater seepage.
(2) Higher moisture content increases compressibility and strain due to reduced effective stress and particle bonding, while higher compaction energy significantly improves stiffness and deformation resistance. Controlling both parameters is essential for long-term stability of GW soil as foundation fill.
(3) A modified permeability model incorporating compaction energy successfully captures the permeability variation. This model offers superior predictive capability and greater practical convenience, as compaction energy is a controllable field parameter, whereas void ratio requires laboratory measurement. The proposed formulation bridges a critical gap between laboratory compaction theory and field quality control.

The findings highlight that, although initial moisture content is secondary to dry density, its influence cannot be neglected. Excess moisture (≥13% in this study) significantly increases soil compressibility, thereby necessitating stricter moisture control during wet-season earthworks. Moreover, this study was conducted under laboratory conditions using standard compaction and oedometer tests. Field-scale effects, such as heterogeneous layering, lateral stress, and long-term wetting–drying cycles, were not considered.
